# Sex and stage-defined exploratory inflammatory and amyloid-associated readout patterns in female APP/PS1 mice exposed to TAK-242

**DOI:** 10.3389/fnagi.2026.1944097

**Published:** 2026-10-14

**Authors:** Xiaolu Ren, Ting Li, Shan Liu, Ling Li, Fang Li

**Affiliations:** 1 Department of Radiology, General Hospital of Ningxia Medical University, Yinchuan, Ningxia, China; 2 School of Health Sciences, Universiti Sains Malaysia, Kubang Kerian, Kelantan, Malaysia; 3 Department of Hematology, General Hospital of Ningxia Medical University, Yinchuan, Ningxia, China; 4 Department of Neurology, General Hospital of Ningxia Medical University, Yinchuan, Ningxia, China

**Keywords:** Alzheimer’s disease, APP/PS1 mouse model, neuroinflammation, TAK-242, toll-like receptor 4 (TLR4)

## Abstract

TLR4-related inflammatory signaling has been implicated in Alzheimer’s disease-related immune responses, and TAK-242 has been used as an experimental tool in studies examining this signaling context. This exploratory study characterized inflammatory cytokine and amyloid-associated histological readout patterns associated with TAK-242 exposure in female early-stage APP/PS1 mice. Female APP/PS1 mice were assigned to saline, lipopolysaccharide, or TAK-242 exposure conditions for 6 weeks, with age-matched non-transgenic mice serving as reference controls (*n* = 8/group). Brain TNF-α, IL-1β, and IL-6 levels were measured by ELISA, and 6E10-positive staining area was quantified by immunohistochemistry. Directional differences in cytokine levels and 6E10-positive staining area were observed between APP/PS1 mice exposed to TAK-242 and untreated APP/PS1 mice. However, because a vehicle-matched APP/PS1 control group was not included, these differences cannot be attributed specifically to TAK-242 and were interpreted as exploratory exposure-associated patterns rather than pharmacological efficacy. This study provides a sex and stage-defined exploratory dataset of inflammatory and amyloid-associated readout patterns in female early-stage APP/PS1 mice to inform future vehicle-controlled investigations.

## Introduction

Alzheimer’s disease is characterized by amyloid-β (Aβ) accumulation, neuronal dysfunction, and sustained innate immune activation (Ogbodo et al., 2022; Heneka et al., 2015). Microglia contribute to cytokine production and Aβ-associated immune responses, representing an important component of disease-related neuroinflammatory processes (Heneka et al., 2015; Reed-Geaghan et al., 2009). However, changes in inflammatory markers do not necessarily indicate corresponding alterations in Aβ processing or disease progression.

Toll-like receptor 4 (TLR4)-related signaling has been implicated in microglial activation and Aβ-associated immune responses in Alzheimer’s disease-related models, although its biological effects appear to be context dependent (Reed-Geaghan et al., 2009; Li et al., 2023; Kawai and Akira, 2010; Tahara et al., 2006; Song et al., 2011; Michaud et al., 2013; Wang et al., 2023; Xu et al., 2023). TAK-242 has been used experimentally in studies examining TLR4-related inflammatory signaling in Alzheimer’s disease models (Zhou et al., 2020; Cui et al., 2020). Previous studies have reported TAK-242-associated alterations in inflammatory activation and amyloid-related readouts in APP/PS1 mice; however, interpretation of these findings requires separation of inflammatory and histological evidence domains (Cui et al., 2020).

Sex and disease stage represent important biological determinants of neuroinflammatory responses and pathological progression in Alzheimer’s disease models. However, quantitative inflammatory and amyloid-associated histological readout patterns following TAK-242 exposure remain insufficiently characterized in female early-stage APP/PS1 mice. Therefore, the present exploratory study provides a sex and stage-defined quantitative assessment of selected inflammatory cytokines and 6E10-positive staining-area readouts in female early-stage APP/PS1 mice under TAK-242 exposure conditions. An LPS-exposed APP/PS1 group was included as an inflammatory-aggravation reference condition to provide contextual comparison for these readouts, rather than to evaluate whether TAK-242 counteracts LPS-induced inflammatory activation. These measurements were evaluated as distinct exploratory biological readouts rather than as direct evidence of TLR4 pathway inhibition or compound-specific pharmacological efficacy. These findings provide exploratory data to inform future vehicle-controlled and pathway-validation studies of selected inflammatory and amyloid-associated readouts in Alzheimer’s disease models.

## Materials and methods

### Animals and experimental design

Female APP/PS1 double-transgenic mice on a C57BL/6 J background (3–4 months old) were obtained from the Experimental Animal Center of Ningxia Medical University and maintained under specific pathogen-free conditions at Ningxia Medical University General Hospital. Age-matched female non-transgenic C57BL/6J mice were used as controls. All experimental procedures were approved by the Ethics Committee of Ningxia Medical University General Hospital, Yinchuan, China (approval no. KYLL-2025-0551), and were conducted in accordance with institutional guidelines.

After acclimatization, mice were randomly assigned to four experimental groups (*n* = 8 per group): non-transgenic control, APP/PS1, APP/PS1 + lipopolysaccharide (LPS), and APP/PS1 + TAK-242. Control and untreated APP/PS1 mice received sterile saline injections. LPS (Sigma-Aldrich, St. Louis, MO, United States) was dissolved in sterile saline and administered intraperitoneally at 1 mg/kg twice weekly for 6 weeks as an inflammatory-aggravation reference condition. TAK-242 (Sigma-Aldrich, St. Louis, MO, United States) was dissolved in 1% dimethyl sulfoxide (DMSO) in double-distilled water and administered intraperitoneally at 10 mg/kg once daily for 6 weeks using a consistent dosing schedule throughout the intervention period.

A vehicle and administration-matched APP/PS1 group receiving 1% DMSO alone according to the same dosing schedule as the TAK-242 group was not included. Therefore, the present design does not allow potential effects of the DMSO vehicle and/or administration regimen to be separated from TAK-242 exposure. Comparisons between untreated APP/PS1 mice and TAK-242-administered mice were consequently interpreted as exposure-associated differences rather than definitive compound-specific pharmacological effects. Following the 6-week intervention period, mice were anesthetized with chloral hydrate (400 mg/kg, intraperitoneally) until deep anesthesia was achieved and subsequently euthanized by cervical dislocation. Hippocampal and cortical tissues were then collected for cytokine measurement and 6E10 immunohistochemical analysis.

### Randomization, blinding, and experimental monitoring

Animals were allocated using a predefined randomization schedule before treatment initiation. The animal was considered the experimental unit for all *in vivo* analyses. No formal *a priori* power calculation was performed because this study was designed as an exploratory study; the sample size (*n* = 8 per group) was selected based on feasibility and previous APP/PS1 pharmacological studies. All animals completed the study and were included in the final analyses. No animals or measurements for the predefined inflammatory cytokine and 6E10-positive staining-area endpoints were excluded from the final analysis.

ELISA samples and immunohistochemical images were coded before analysis. ImageJ-based quantification of 6E10-positive staining area was performed by investigators blinded to group allocation. Animals were monitored throughout the treatment period for body weight changes, general condition, mobility, and injection-related abnormalities.

### Cytokine measurement by ELISA

Brain TNF-α, IL-1β, and IL-6 levels were quantified using commercially available mouse ELISA kits according to the manufacturers’ protocols. Hippocampal and cortical tissues were homogenized in ice-cold extraction buffer and centrifuged, and the resulting supernatants were used for cytokine determination. Cytokine concentrations were normalized to total protein content and expressed as pg/mg protein. All samples and standards were analyzed in duplicate, and concentrations were calculated using plate-specific standard curves. Sample identifiers were coded before measurement, and assay records were retained for quality verification. During revision, the archived laboratory and procurement records available from the original experiments were re-reviewed. However, the original ELISA kit catalog numbers could not be verified with sufficient confidence and were therefore not retrospectively reconstructed.

The archived laboratory records were re-reviewed during revision; however, the exact 6E10 antibody catalog number, working dilution, section thickness, and several protocol-specific incubation conditions could not be verified with sufficient confidence. These details were therefore not retrospectively inferred or reconstructed.

### 6E10 immunohistochemistry and ImageJ-based quantification

6E10 immunohistochemistry was performed in predefined hippocampal and cortical regions using consistent imaging parameters across experimental groups. Images were coded before analysis, and ImageJ-based quantification was performed by an investigator blinded to experimental allocation. For each animal, five non-overlapping fields from comparable anatomical regions were analyzed and averaged to generate a single animal-level measurement. A consistent thresholding strategy was applied within the same analytical batch without group-specific manual adjustment. The outcome was defined as the percentage of 6E10-positive staining area within the predefined region of interest.

The 6E10 measurement was interpreted as an immunohistochemical staining-area readout rather than as a comprehensive measure of amyloid pathology. Plaque number, plaque-size distribution, integrated optical density, amyloid-beta species composition, and amyloid-beta production or clearance processes were not assessed. During revision, the archived experimental records were re-reviewed; however, the original 6E10 antibody catalog number, working dilution, section thickness, and several protocol-specific incubation conditions could not be verified with sufficient confidence. These details were therefore not retrospectively inferred or reconstructed.

### Statistical analysis

Data are presented as mean ± standard deviation (SD). The animal was used as the experimental unit for all statistical analyses. For 6E10 immunohistochemistry, multiple field-level measurements from each animal were averaged before group-level statistical testing.

Normality was assessed using the Shapiro–Wilk test, and homogeneity of variance was evaluated using Levene’s test. When normality and variance assumptions were satisfied, one-way analysis of variance (ANOVA) followed by Tukey’s honestly significant difference (HSD) *post hoc* test was applied. When variance heterogeneity was detected, Welch’s ANOVA followed by Games–Howell *post hoc* testing was used.

Because this study was designed as an exploratory Brief Research Report with predefined biological readouts, statistical findings were interpreted descriptively rather than as confirmatory evidence. For the principal comparison between untreated APP/PS1 and APP/PS1 + TAK-242 groups, the magnitude of group differences was additionally quantified using Hedges’ g with 95% confidence intervals. Morris water maze and Y-maze measures were treated as secondary exploratory outcomes because they were not prespecified as primary efficacy endpoints and were not accompanied by the full set of behavioral control measures required for definitive interpretation of cognitive performance. Detailed descriptive statistics, assumption testing results, global comparisons, effect-size estimates, and selected pairwise comparisons are provided in the Supplementary materials.

## Results

### Main inflammatory and 6E10 staining-area readouts

Group-level analyses identified differences among experimental groups in the predefined main readouts, including 6E10-positive staining area, TNF-α, IL-1β, and IL-6 (all global *p* < 0.001; Figure 1, Table 1, and Supplementary Table S1). APP/PS1 mice showed higher 6E10-positive staining area and pro-inflammatory cytokine levels than non-transgenic controls. LPS-treated APP/PS1 mice exhibited further increases in these readouts compared with untreated APP/PS1 mice. Because an *in vivo* APP/PS1 + LPS + TAK-242 co-administration group was not included, the LPS group was not used to evaluate TAK-242-associated effects under inflammatory challenge conditions.

**Figure 1:**
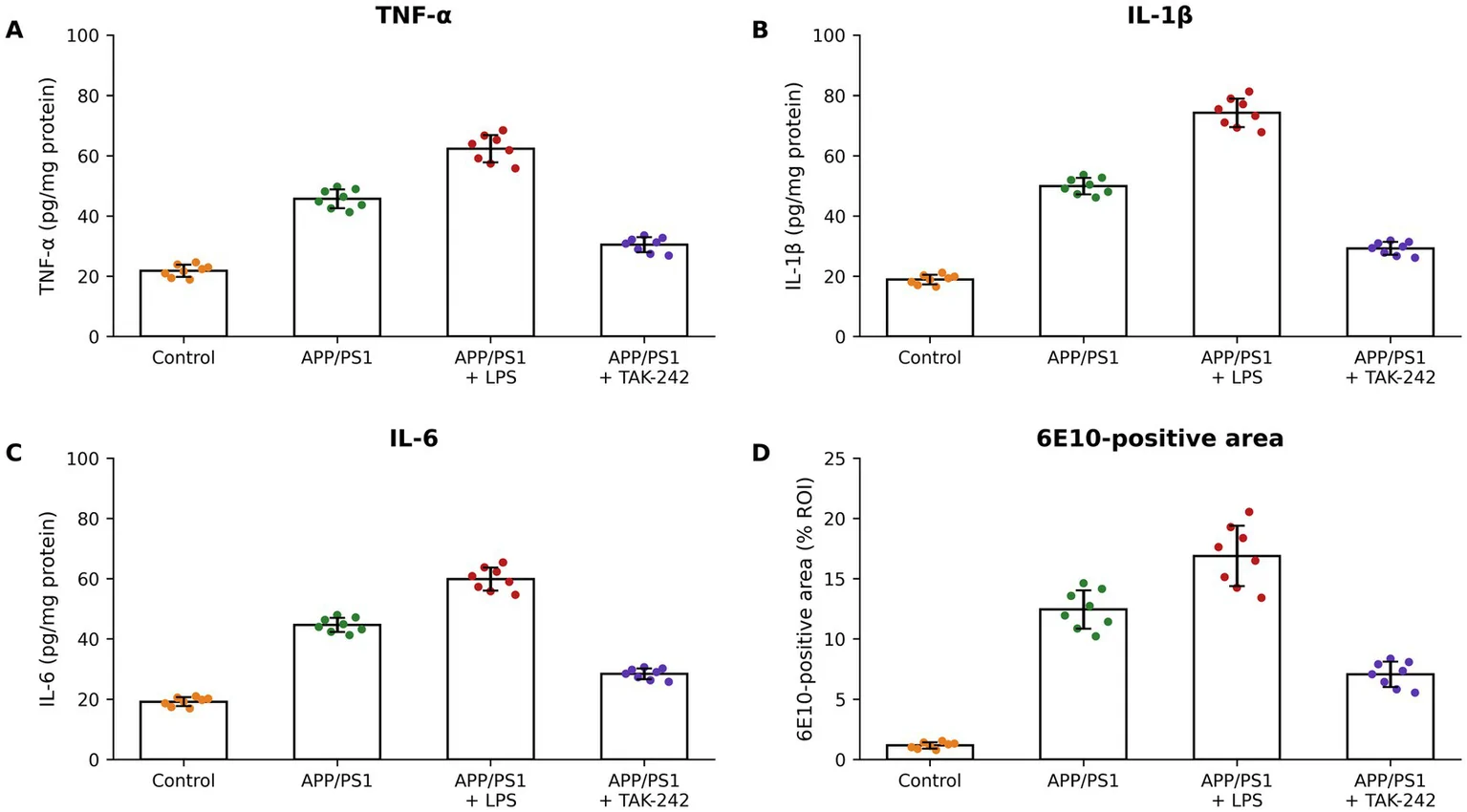
Inflammatory cytokine and 6E10-positive staining-area readouts across experimental groups. **(A)**, TNF-α. **(B)**, IL-1β. **(C)**, IL-6. **(D)**, 6E10-positive staining area (% ROI). Bars represent mean ± SD; dots represent individual animals; *n* = 8 per group. The LPS group served as an inflammatory-aggravation reference, and 6E10-positive area was interpreted only as a staining-area readout.

**Table 1:** Exploratory inflammatory and 6E10 immunohistochemical readouts across experimental groups.

Group	6E10-positive staining area (% ROI)	TNF-α (pg/mg protein)	IL-1β (pg/mg protein)	IL-6 (pg/mg protein)
Control	1.2 ± 0.3	21.8 ± 2.0	18.9 ± 1.6	19.2 ± 1.4
APP/PS1	12.4 ± 1.6	45.7 ± 3.1	49.9 ± 2.7	44.6 ± 2.4
APP/PS1 + LPS	16.9 ± 2.5	62.3 ± 4.6	74.3 ± 4.7	59.9 ± 3.9
APP/PS1 + TAK-242	7.1 ± 1.1	30.5 ± 2.5	29.3 ± 2.2	28.4 ± 1.8

Values are presented as mean ± SD (*n* = 8 per group). LPS, lipopolysaccharide; ROI, region of interest; SD, standard deviation. The 6E10-positive staining area was interpreted as an immunohistochemical readout rather than a direct measure of amyloid-beta burden or amyloid processing.

Descriptive comparisons identified differences in 6E10-positive staining area and TNF-α, IL-1β, and IL-6 levels between TAK-242-administered and untreated APP/PS1 mice (Supplementary Table S2). For the principal comparison between untreated APP/PS1 and APP/PS1 + TAK-242 groups, Hedges’ g estimates for all four predefined readouts, together with corresponding 95% confidence intervals, are provided in Supplementary Table S2. These effect-size estimates quantify the magnitude of the observed exposure-associated differences but should not be interpreted as evidence of TAK-242-specific pharmacological effects or efficacy because a vehicle and administration-matched APP/PS1 control group was not included. These readouts remained above those observed in non-transgenic controls.

The 6E10 endpoint was quantified as the percentage of positive staining area within predefined regions of interest and was interpreted as an immunohistochemical staining-area readout. This measurement was not considered a direct assessment of amyloid-beta burden, plaque number, plaque-size distribution, Aβ species composition, or Aβ production/clearance processes.

Exploratory behavioral observations from the Morris water maze and Y-maze tests are provided in Supplementary Figure S1 and Supplementary Tables S4, S5. These task-performance measures were not predefined as primary outcomes and were not accompanied by the full set of behavioral control measures required for definitive cognitive interpretation. They were therefore interpreted as exploratory observations rather than evidence of cognitive efficacy.

## Discussion

This Brief Research Report provides a focused assessment of inflammatory cytokine and amyloid-associated histological readout patterns in female early-stage APP/PS1 mice associated with TAK-242 exposure conditions. A key feature of this study is the separation of inflammatory, histological, and behavioral observations into distinct evidentiary layers, recognizing that these measures represent related but non-equivalent aspects of Alzheimer’s disease-related pathology. Alzheimer’s disease involves complex interactions among amyloid-associated changes, neuronal dysfunction, and innate immune activation, with neuroinflammatory processes representing an important component of disease progression rather than an isolated therapeutic target (Ogbodo et al., 2022; Heneka et al., 2015).

The observed readout patterns showed similar directional trends across TNF-α, IL-1β, IL-6, and 6E10-positive staining area, with differences observed between APP/PS1 mice exposed to TAK-242 and untreated APP/PS1 mice. However, the TAK-242-treated group received 1% DMSO, whereas the untreated APP/PS1 group did not receive a DMSO-matched vehicle regimen. Because an APP/PS1 vehicle-control group receiving 1% DMSO according to the same administration schedule was not included, potential effects attributable to the vehicle and/or administration regimen cannot be excluded. Accordingly, the observed differences cannot be attributed specifically to TAK-242 and should be interpreted only as exploratory exposure-associated patterns rather than evidence of TAK-242-specific pharmacological activity. Future studies should incorporate a vehicle and administration-matched APP/PS1 control group to distinguish TAK-242-specific effects from vehicle or procedure-related effects. This cautious interpretation is consistent with previous studies demonstrating that TLR4-associated signaling influences Alzheimer’s disease-related immune responses, while outcomes may vary depending on experimental models and biological contexts (Reed-Geaghan et al., 2009; Tahara et al., 2006; Song et al., 2011).

The present findings are broadly consistent with previous studies showing that TLR4-related innate immune pathways participate in microglial activation, Aβ-associated immune responses, and inflammatory signaling (Reed-Geaghan et al., 2009; Kawai and Akira, 2010). Experimental modulation of TLR4 signaling has been associated with altered microglial states and Alzheimer’s disease-related pathological features; however, the magnitude and direction of these effects differ across disease models and intervention strategies (Song et al., 2011; Michaud et al., 2013; Cui et al., 2020). The present study was not designed as a pathway-validation investigation, and TLR4 expression and downstream signaling components, including MyD88 and NF-κB activation, were not directly assessed. Accordingly, the present findings should not be interpreted as evidence of TLR4 pathway engagement, inhibition, or mechanistic specificity. In addition, the inflammatory assessment was limited to TNF-α, IL-1β, and IL-6, without direct evaluation of microglial or astrocytic activation using markers such as Iba1 or GFAP. Therefore, these cytokine measurements represent selected inflammatory readouts rather than a comprehensive assessment of neuroinflammation or glial responses. Accordingly, the observed changes should be interpreted as TAK-242 exposure-associated patterns rather than direct evidence of TLR4 pathway inhibition or a TLR4-specific causal mechanism.

The 6E10-positive staining-area measurement should be interpreted as a coarse histological readout rather than a comprehensive measure of amyloid pathology or a direct indicator of Aβ clearance or altered amyloid processing. Staining-area quantification does not provide information on plaque number, plaque-size distribution, Aβ species composition, or Aβ production and clearance. Although TLR-related signaling has been linked to Aβ uptake, deposition, and immune-mediated responses (Tahara et al., 2006; Song et al., 2011), staining-area quantification alone cannot distinguish among different amyloid-related biological processes. Further studies incorporating biochemical amyloid quantification, plaque characterization, and pathway-level analyses will be required to determine the biological basis underlying differences in 6E10-positive staining area.

Several limitations should be considered. Most importantly, the absence of a vehicle-matched APP/PS1 control group prevents separation of TAK-242-specific effects from potential effects related to the 1% DMSO vehicle or repeated injection procedures. The exploratory sample size, inclusion of only female mice at a single early disease stage, evaluation of only one TAK-242 dose and one treatment duration, and lack of molecular pathway validation further restrict the generalizability and interpretation of the findings. Accordingly, the present findings should not be generalized to male animals, later disease stages, or alternative TAK-242 dosing regimens without further investigation. In particular, TLR4 expression and downstream signaling molecules such as MyD88 and NF-κB were not assessed; therefore, the present data cannot establish direct inhibition of the TLR4 signaling pathway. In addition, inflammatory assessment was limited to TNF-α, IL-1β, and IL-6, without evaluation of microglial or astrocytic activation markers such as Iba1 or GFAP. Thus, the present cytokine data should be regarded as selected inflammatory readouts rather than a comprehensive characterization of neuroinflammation. Certain reagent and protocol-specific historical details could not be retrospectively verified from the available experimental records, which may limit complete procedural reproducibility. Because the behavioral measures were not prespecified as primary efficacy outcomes and key control measures for definitive cognitive interpretation were unavailable, the Morris water maze and Y-maze findings should be regarded as descriptive exploratory observations only. Future investigations incorporating vehicle-controlled experimental designs, microglial phenotyping, pathway-specific validation, and comprehensive behavioral assessment are warranted to further clarify the biological significance of TAK-242-associated readout patterns in Alzheimer’s disease models.

## Conclusion

In female early-stage APP/PS1 mice, TNF-α, IL-1β, IL-6, and 6E10-positive staining-area readouts differed across experimental conditions, including directional differences between TAK-242-exposed and untreated APP/PS1 mice. Because a vehicle and administration-matched APP/PS1 control group was not included, these differences cannot be attributed specifically to TAK-242. Moreover, TLR4 expression and downstream signaling components, including MyD88 and NF-κB activation, were not directly assessed; therefore, the findings should not be interpreted as direct evidence of TLR4 pathway inhibition. Overall, the results should be regarded as exploratory exposure-associated patterns specific to female mice at an early disease stage.

## Data Availability

The original contributions presented in the study are included in the article/Supplementary material, further inquiries can be directed to the corresponding author.
