# Supplementary material for: Sex and stage-defined exploratory inflammatory and amyloid-associated readout patterns in female APP/PS1 mice exposed to TAK-242
Source: Front Aging Neurosci. 2026 Oct 14;18:1944097. doi: 10.3389/fnagi.2026.1944097 (PMC13713749; doi:10.3389/fnagi.2026.1944097)
Supplement: Supplementary file 1 [file Data_Sheet_1.docx]

**Supplementary Material**

This supplementary material provides descriptive statistics, assumption checks, global tests, and selected pairwise comparisons for the predefined main readouts, together with descriptive summaries of secondary exploratory task-performance observations.

**Supplementary Table S1. Assumption checks and global tests for main readouts**

| **Endpoint** | **Minimum Shapiro–Wilk P value across groups** | **Levene P** | **Selected global test** | **Global statistic** | **df** | **Global P** | **Post hoc** |
| --- | --- | --- | --- | --- | --- | --- | --- |
| 6E10-positive staining area (% ROI) | 0.562 | <0.001 | Welch ANOVA | 267.522 | 3, 12.361 | <0.001 | Games-Howell |
| TNF-α (pg/mg protein) | 0.525 | 0.040 | Welch ANOVA | 218.837 | 3, 15.100 | <0.001 | Games-Howell |
| IL-1β (pg/mg protein) | 0.500 | 0.006 | Welch ANOVA | 463.862 | 3, 14.854 | <0.001 | Games-Howell |
| IL-6 (pg/mg protein) | 0.552 | 0.011 | Welch ANOVA | 379.405 | 3, 14.953 | <0.001 | Games-Howell |

Normality was assessed using Shapiro-Wilk tests within each group; homogeneity of variance was assessed using mean-centered Levene's test. Levene P values smaller than 0.001 are reported as P < 0.001. Welch ANOVA and Games-Howell post hoc testing were used for predefined main readouts to provide a conservative and consistent analytical framework across cytokine and 6E10-positive staining-area readouts in the presence of variance heterogeneity. Because Welch ANOVA was used for the predefined main readouts, conventional eta-squared values were not emphasized in this table.

**Supplementary Table S2. Selected APP/PS1 versus APP/PS1 + TAK-242 comparisons for predefined main readouts**

| **Endpoint** | **Mean difference (TAK-242 − APP/PS1)** | **95% CI lower** | **95% CI upper** | **Adjusted P (Games–Howell)** | **Hedges’ g** | **95% CI for Hedges’ g** |
| --- | --- | --- | --- | --- | --- | --- |
| 6E10-positive staining area (% ROI) | −5.379 | −7.381 | −3.376 | <0.001 | **−3.763** | **−5.467 to −2.059** |
| TNF-α (pg/mg protein) | −15.221 | −19.338 | −11.104 | <0.001 | **−5.113** | **−7.245 to −2.980** |
| IL-1β (pg/mg protein) | −20.662 | −24.257 | −17.068 | <0.001 | **−7.948** | **−11.051 to −4.845** |
| IL-6 (pg/mg protein) | −16.227 | −19.314 | −13.141 | <0.001 | **−7.284** | **−10.155 to −4.414** |

These pairwise comparisons are provided as exploratory supportive analyses. Hedges’ g was calculated for the predefined comparison between untreated APP/PS1 and APP/PS1 + TAK-242 groups, with negative values indicating lower readout levels in the APP/PS1 + TAK-242 group. The corresponding 95% confidence intervals are provided to facilitate interpretation of the magnitude and precision of the observed differences. Because a vehicle- and administration-matched APP/PS1 control group was not included, these effect-size estimates should be interpreted as quantifying exposure-associated differences rather than definitive TAK-242-specific pharmacological effects.

**Supplementary Table S3. Descriptive statistics for predefined main readouts**

| **Endpoint** | **Group** | **n** | **Mean** | **SD** | **SEM** | **Min** | **Max** |
| --- | --- | --- | --- | --- | --- | --- | --- |
| 6E10-positive staining area (% ROI) | Control | 8 | 1.169 | 0.261 | 0.092 | 0.79 | 1.54 |
| 6E10-positive staining area (% ROI) | APP/PS1 | 8 | 12.438 | 1.595 | 0.564 | 10.21 | 14.62 |
| 6E10-positive staining area (% ROI) | APP/PS1 + LPS | 8 | 16.886 | 2.510 | 0.887 | 13.41 | 20.54 |
| 6E10-positive staining area (% ROI) | APP/PS1 + TAK-242 | 8 | 7.059 | 1.053 | 0.372 | 5.53 | 8.35 |
| TNF-α (pg/mg protein) | Control | 8 | 21.841 | 2.023 | 0.715 | 18.91 | 24.62 |
| TNF-α (pg/mg protein) | APP/PS1 | 8 | 45.693 | 3.114 | 1.101 | 41.28 | 49.74 |
| TNF-α (pg/mg protein) | APP/PS1 + LPS | 8 | 62.335 | 4.551 | 1.609 | 55.83 | 68.47 |
| TNF-α (pg/mg protein) | APP/PS1 + TAK-242 | 8 | 30.471 | 2.480 | 0.877 | 26.86 | 33.57 |
| IL-1β (pg/mg protein) | Control | 8 | 18.900 | 1.595 | 0.564 | 16.53 | 21.17 |
| IL-1β (pg/mg protein) | APP/PS1 | 8 | 49.919 | 2.721 | 0.962 | 46.15 | 53.68 |
| IL-1β (pg/mg protein) | APP/PS1 + LPS | 8 | 74.290 | 4.736 | 1.674 | 67.84 | 81.26 |
| IL-1β (pg/mg protein) | APP/PS1 + TAK-242 | 8 | 29.256 | 2.163 | 0.765 | 26.14 | 31.86 |
| IL-6 (pg/mg protein) | Control | 8 | 19.196 | 1.431 | 0.506 | 16.97 | 20.93 |
| IL-6 (pg/mg protein) | APP/PS1 | 8 | 44.611 | 2.361 | 0.835 | 41.24 | 47.91 |
| IL-6 (pg/mg protein) | APP/PS1 + LPS | 8 | 59.859 | 3.851 | 1.362 | 54.62 | 65.38 |
| IL-6 (pg/mg protein) | APP/PS1 + TAK-242 | 8 | 28.384 | 1.816 | 0.642 | 25.73 | 30.58 |

Values are presented as descriptive statistics. Mean, SD, and SEM are reported to three decimal places; minimum and maximum values are reported to two decimal places, consistent with the precision of the original records. n indicates the number of animals per group.

**Supplementary Table S4. Descriptive statistics for secondary exploratory task-performance observations**

| **Endpoint** | **Group** | **n** | **Mean** | **SD** | **SEM** | **Min** | **Max** |
| --- | --- | --- | --- | --- | --- | --- | --- |
| MWM final acquisition-day latency (s) | Control | 8 | 18.871 | 2.720 | 0.962 | 14.95 | 22.74 |
| MWM final acquisition-day latency (s) | APP/PS1 | 8 | 35.983 | 3.395 | 1.200 | 31.04 | 40.25 |
| MWM final acquisition-day latency (s) | APP/PS1 + LPS | 8 | 42.084 | 3.969 | 1.403 | 36.58 | 47.82 |
| MWM final acquisition-day latency (s) | APP/PS1 + TAK-242 | 8 | 26.091 | 2.557 | 0.904 | 22.61 | 29.48 |
| Y-maze spontaneous alternation (%) | Control | 8 | 71.422 | 3.705 | 1.310 | 66.17 | 76.82 |
| Y-maze spontaneous alternation (%) | APP/PS1 | 8 | 49.136 | 3.381 | 1.195 | 44.29 | 53.85 |
| Y-maze spontaneous alternation (%) | APP/PS1 + LPS | 8 | 42.859 | 4.564 | 1.613 | 36.93 | 49.64 |
| Y-maze spontaneous alternation (%) | APP/PS1 + TAK-242 | 8 | 63.351 | 4.193 | 1.482 | 57.96 | 68.92 |

Values are presented as descriptive statistics. Mean, SD, and SEM are reported to three decimal places; minimum and maximum values are reported to two decimal places, consistent with the precision of the original records. n indicates the number of animals per group.

**Supplementary Table S5. Descriptive summary of secondary exploratory task-performance observations**

| **Endpoint** | **APP/PS1** | **APP/PS1 + TAK-242** | **Descriptive difference** |
| --- | --- | --- | --- |
| MWM final acquisition-day latency (s) | 35.983 ± 3.395 | 26.091 ± 2.557 | Lower latency under the TAK-242 administration condition |
| Y-maze spontaneous alternation (%) | 49.136 ± 3.381 | 63.351 ± 4.193 | Higher spontaneous alternation under the TAK-242 administration condition |

**Note**: Values are mean ± SD. These secondary task-performance observations are descriptive only and were not prespecified efficacy outcomes. They were not used to infer cognitive or memory-related improvement because key behavioral control measures were unavailable.

**Supplementary Figure S1. Secondary exploratory task-performance observations**

Supplementary Figure S1. Secondary exploratory task-performance observations. A, Morris water maze final acquisition-day latency. B, Y-maze spontaneous alternation. Bars represent mean ± SD; dots represent individual animals (n = 8 per group). These exploratory behavioral observations were interpreted descriptively and were not considered primary efficacy outcomes.
